# Design and fabrication of recombinant reflectin-based multilayer reflectors: bio-design engineering and photoisomerism induced wavelength modulation

**DOI:** 10.1038/s41598-021-94042-6

**Published:** 2021-07-16

**Authors:** Emmanuel Wolde-Michael, Aled D. Roberts, Derren J. Heyes, Ahu G. Dumanli, Jonny J. Blaker, Eriko Takano, Nigel S. Scrutton

**Affiliations:** 1 Department of Chemistry, EPSRC/BBSRC Future Biomanufacturing Research Hub, Manchester Institute of Biotechnology, The University of Manchester, Manchester, M1 7DN UK; 2 Department of Materials and Henry Royce Institute, The University of Manchester, Manchester, M13 9PL UK

**Keywords:** Bioinspired materials, Biophysics

## Abstract

The remarkable camouflage capabilities of cephalopods have inspired many to develop dynamic optical materials which exploit certain design principles and/or material properties from cephalopod dermal cells. Here, the angle-dependent optical properties of various single-layer reflectin thin-films on Si wafers are characterized within the UV–Vis–NIR regions. Following this, initial efforts to design, fabricate, and optically characterize a bio-inspired reflectin-based multilayer reflector is described, which was found to conserve the optical properties of single layer films but exhibit reduced angle-dependent visible reflectivity. Finally, we report the integration of phytochrome visible light-induced isomerism into reflectin-based films, which was found to subtly modulate reflectin thin-film reflectivity.

## Introduction

**Figure 1:**
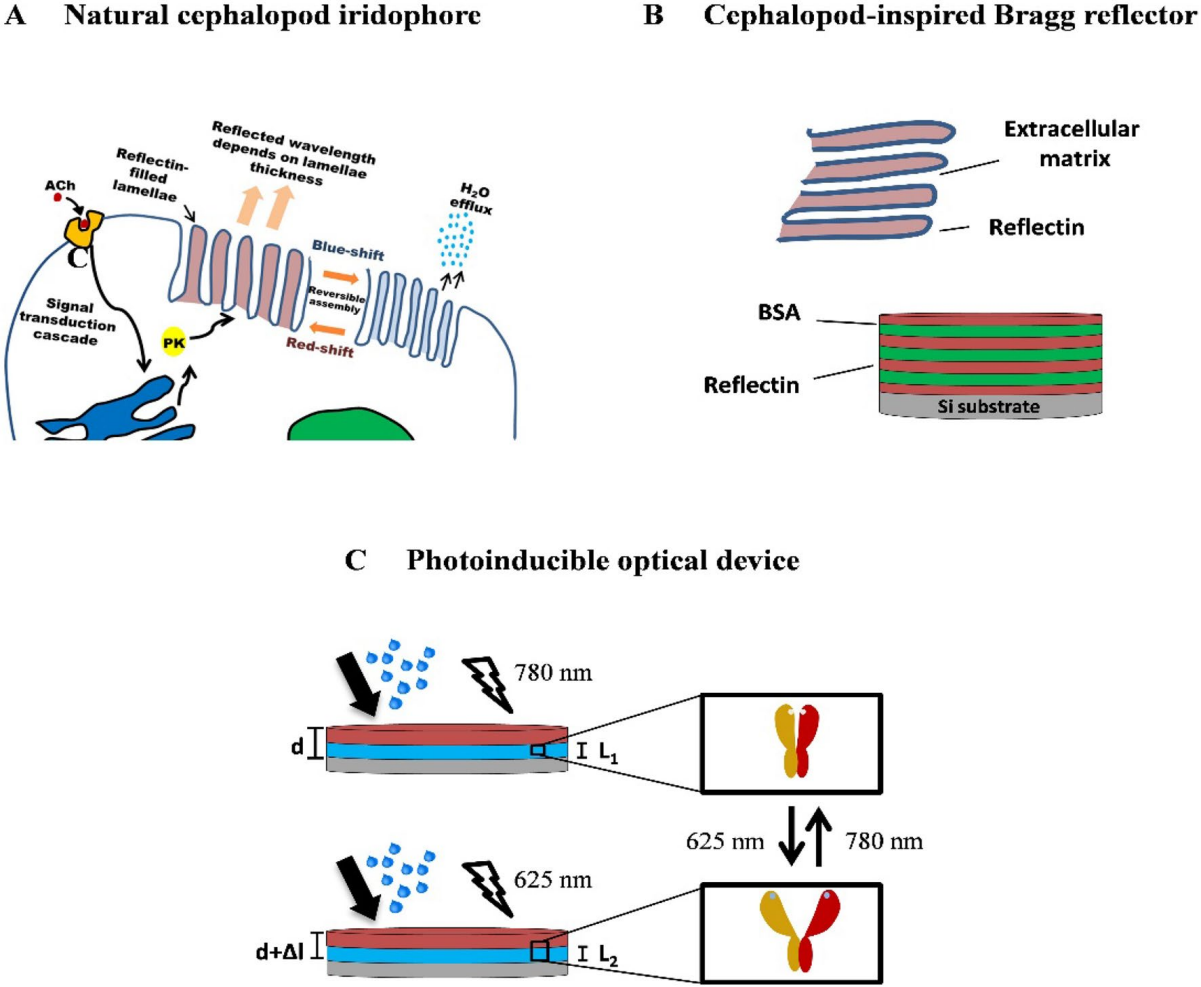
(**A**) Schematic figure showing cephalopod iridophore neurochemical activation^9^. (**B**) Schematic figure of cephalopod-inspired reflectin/BSA Bragg reflector alongside the iridophore Bragg reflector. (**C**) Schematic figure of reflectin-phytochrome device switching between two states. Upon illumination with red/far-red light in the presence of water vapor the phytochrome can convert between an ‘open’ and ‘closed’ state.

Reflectins are a unique family of high-refractive index proteins native to cephalopods (squid, octopus, and cuttlefish)^1^. Consisting of repeating motifs (rich in aromatic and sulfur-containing amino acids) separated by positively charged linkers, these proteins have been found within cephalopod chromophores in various configurations, such as distributed within sheath cells surrounding pigment-filled chromatocytes^2^, and forming polydispersed microspheres within leucocytes^3^—producing a scattering effect in both cases. These proteins, however, are more commonly associated with cellular Bragg reflectors known as iridophores, where intracellular lamellae containing reflectins are spatially separated by the extracellular matrix (Fig. 1A)^4^. Iridescence in this case arises from coherent Bragg reflection from successive layers, defined by interference^5^. Some cephalopods, such as squid in the *Loligidae* family, are able to tightly control the optical properties of these iridophores using a neurotransmitter, acetylcholine (ACh)^6^. ACh triggers a signal-transduction cascade which leads to the phosphorylation of reflectins, reducing net charge and triggering reversible hierarchical assembly into a more condensed structure^7,8^. Reduced ion exposure then leads to lamellae dehydration, further increasing the refractive index contrast^9^. Collectively, along with chromatophores and leucophores, iridophores endow cephalopods with a high level of control over their body coloration and patterning, which they use for both camouflage and signaling^1^.

Since their discovery, researchers have been working towards the development of dynamic, optically active camouflage technologies which exploit the properties of recombinant reflectins^9–14^. Facilitating this, recent efforts have been directed toward the characterization of reflectins in vitro, revealing properties such as pH-dependent particle-size formation^15^, the differential roles of motifs and linker regions^8^, the role of key amino-acid residues on assembly^16^, reflectin conductive properties^17,18^, and the effect of small molecules on higher-order assembly^19^. Advances in the design, fabrication, and characterization of reflectin-based materials have revealed properties such as thickness-dependent coloration^11–13,20,21^, broad near-infra-red (NIR) reflectance^13^, and induced light scattering^23,24^. It has also been demonstrated that the thickness (and therefore optical properties) of these materials can be controlled both pre-fabrication, by varying parameters such as flow-coating angle and sample concentration^20,21^, and post-fabrication, via vapor-induced swelling^20–22^, applying uniaxial strain^12^, or proton conduction^11^. As most reflectin-based materials are fabricated in the form of thin-films/coatings, their optical reflectivity $\left( \lambda \right)$ depends on both the layer thickness (*d*) and the angle of incidence $\left( \theta \right)$, as shown in Eq. (1), where *n* is average refractive index and *m* is order of reflection. However, while significant efforts have been directed towards developing novel methods of controlling reflectin layer thickness to modulate the optical properties of these materials, few studies have characterized and/or modified the angle-dependent reflectance of such films^23^.

1 $$2nd\;cos\theta = \left( {m + \frac{1}{2}} \right)\lambda \quad m = 0, \pm 1, \pm 2, \ldots$$

Here, the specular reflectance of a range of reflectin single-layer thin-films on Si wafers have been characterized over a wide wavelength range (185–3300 nm), revealing previously unreported optical properties. The angle-dependent reflectance of these films was then subsequently characterized by varying the angle of incidence between 20° and 70°, revealing significant spectral changes. Following this, we describe our initial efforts to design and fabricate a cephalopod-inspired reflectin-based multilayer reflector (Fig. 1B), exhibiting reduced angle-dependent visible reflectivity. Finally, we introduce a novel method of subtly shifting thin-film optical properties via visible light-induced isomerism (Fig. 1C).

## Results and discussion

**Figure 2:**
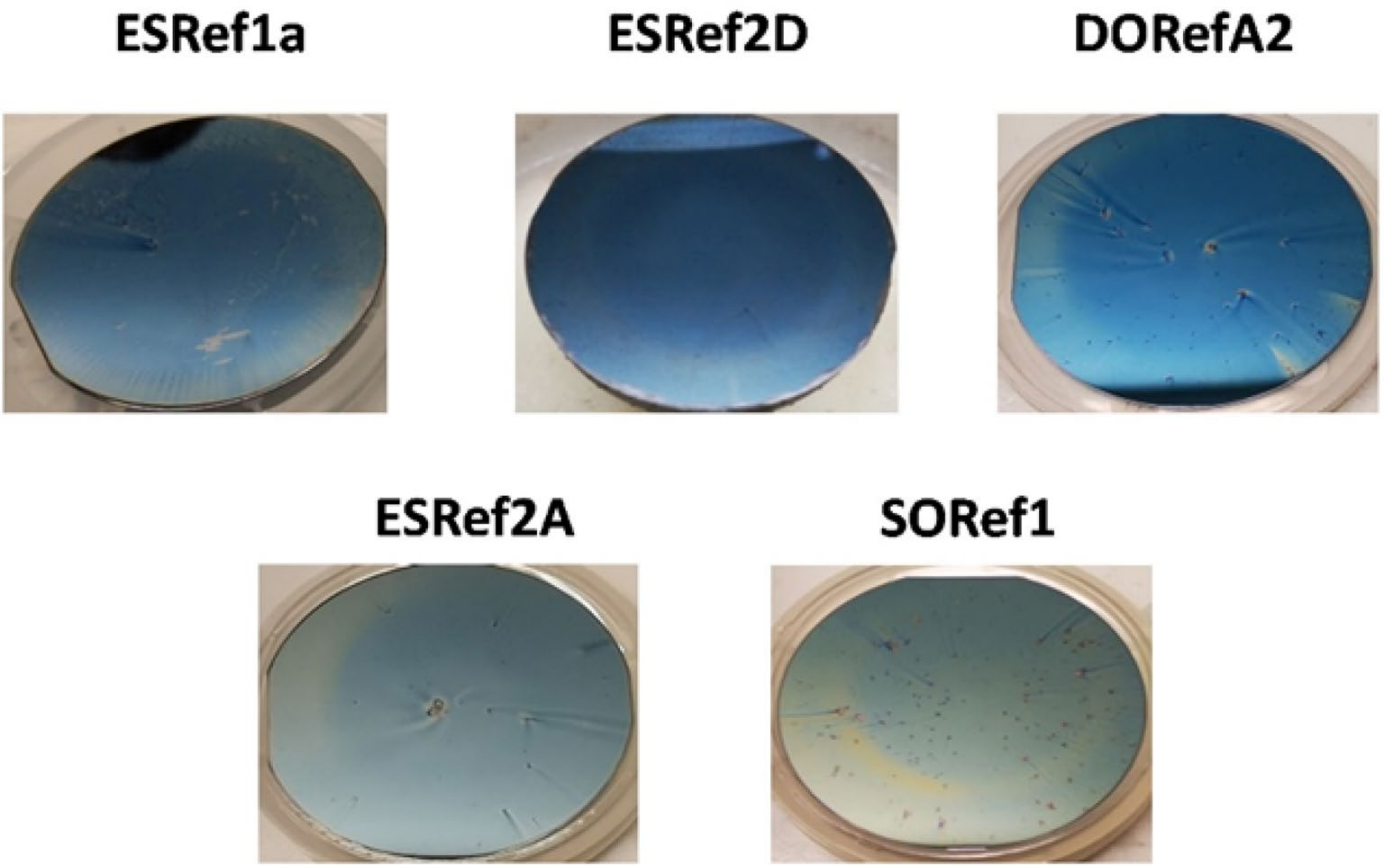
Optical images of reflectin thin-films following fabrication by spin coating 1% w/w reflectin in HFIP onto a clean Si wafer. Camera images were taken following drying. DORefA2/BSA multilayer films were fabricated by spin coating reflectin and BSA sequentially.

**Figure 3:**
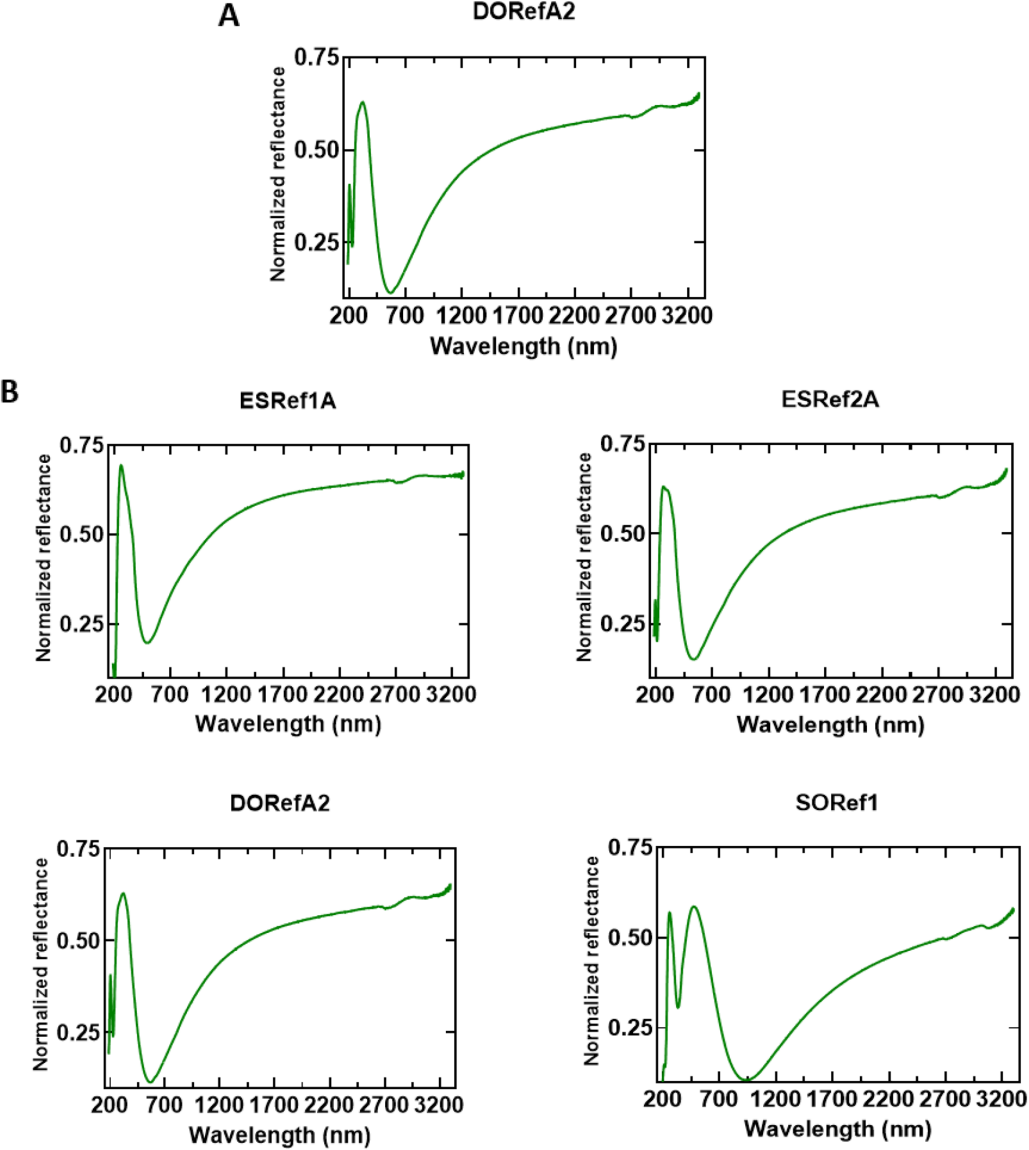
(**A**) UV–Vis–NIR reflectance spectra (200–3300 nm) of DORefA2 single-layer film fabricated by spin coating 1% w/w reflectin DORefA2 in HFIP onto clean Si wafers. (**B**) UV–Vis–NIR reflectance spectra (200–3300 nm) of other single-layer films fabricated under the same conditions. Angle of incidence: 20°.

**Figure 4:**
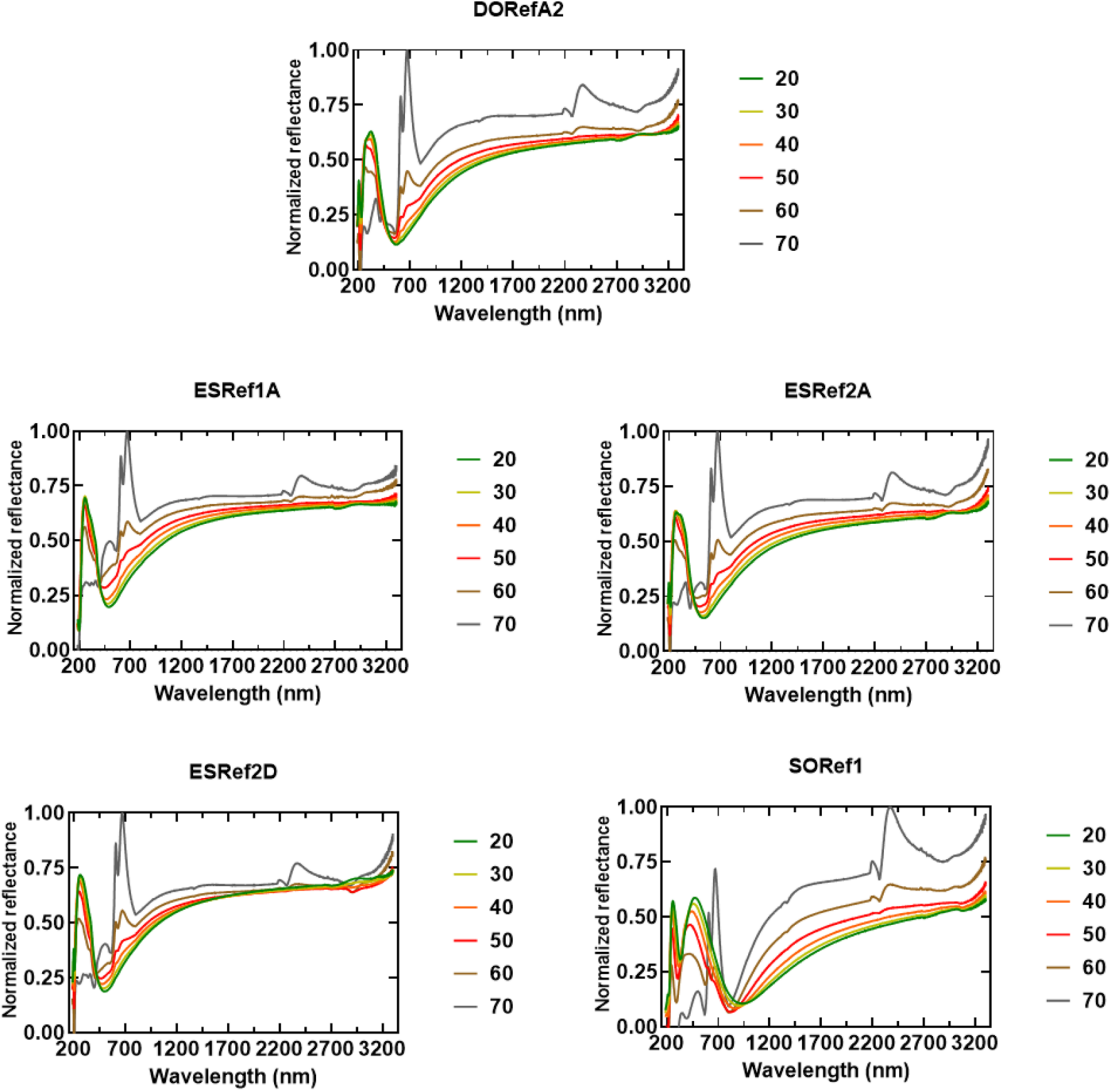
UV–Vis–NIR reflectance spectra (200–3300 nm) of single-layer films fabricated by spin coating 1% w/w reflectin in HFIP onto clean Si wafers. The angle of incidence was varied between 20° and 70° (10° intervals).

**Figure 5:**
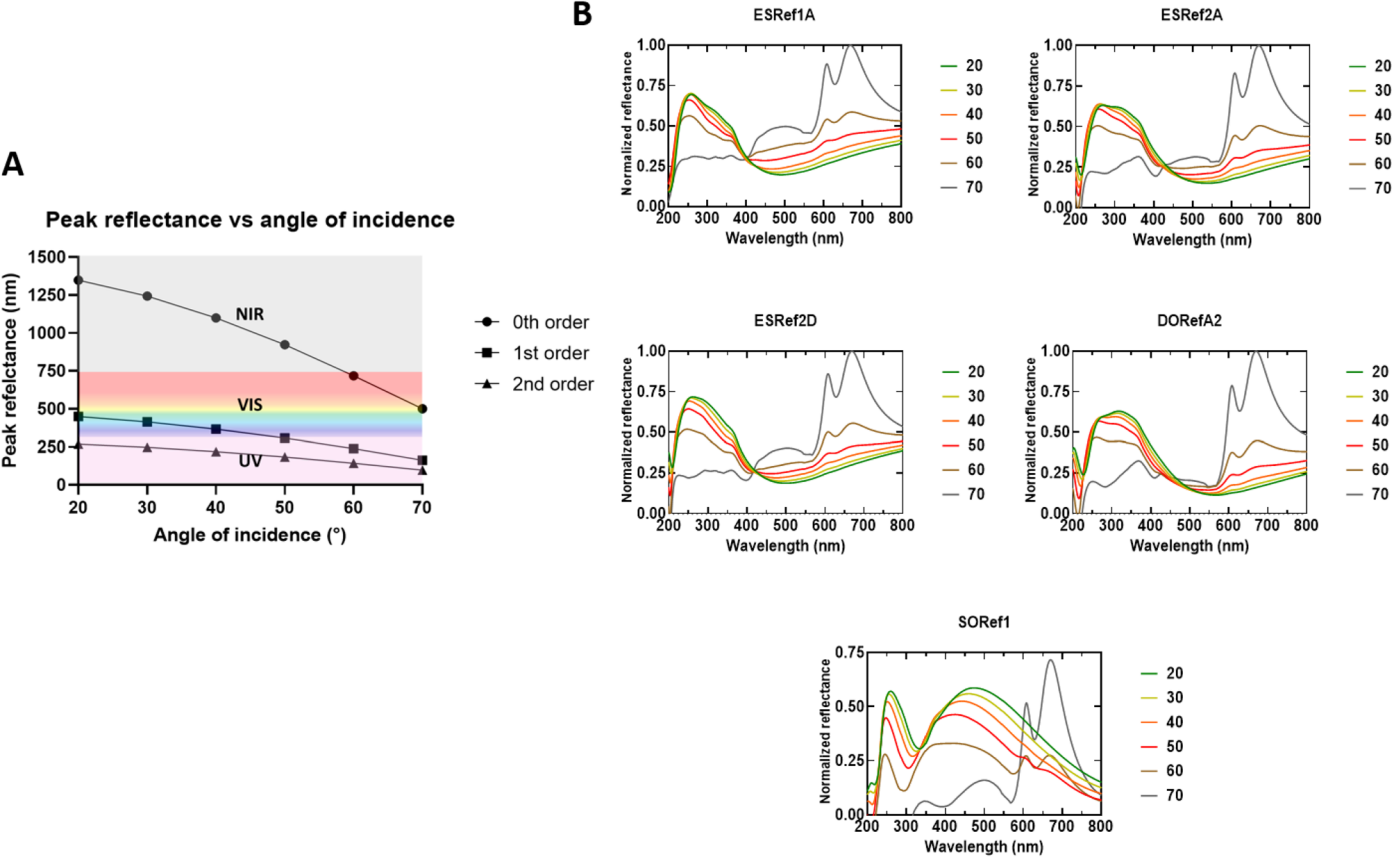
(**A**) Calculated peak reflectance of 0th, 1st, and 2nd order of reflection vs angle of incidence as calculated using Eq. (1). (**B**) UV–VIS reflectance spectra (200–800 nm) of single-layer films fabricated by spin coating 1% w/w reflectin in HFIP onto clean Si wafers. The angle of incidence was varied between 20° and 70° (10° intervals).

All reflectin isoforms (referred to as XXRefYY, where XX is the origin, and YY is the reflectin isoform, Figure S1) were expressed in *Escherichia coli* from recombinant plasmids and were found to be sequestered in inclusion bodies as previously reported^15,21^. Inclusion bodies were isolated using standard inclusion body preparations^24^, solubilized under strongly denaturing conditions, and purified using high pressure liquid chromatography (HPLC, Figure S2). Pure fractions were combined before being lyophilized and stored at 4 °C, and purity was confirmed by SDS PAGE (Figure S3)^25^. Upon spin-coating onto clean Si wafers under the same conditions, all single-layer films appeared blue under ambient light, which suggests comparable thicknesses were achieved. This blue color suggests samples have peak reflectance in the visible region between 380 and 450 nm, although two samples, SORef1 in particular, appear cyan colored, suggesting peak reflectance may be slightly red-shifted^5^ (Fig. 2). Si wafers were selected due to their highly reflective surface, leading to optical responses more representative of thin-film reflectors when compared to substrates such as glass (Figure S4). Using scanning electron microscopy (SEM), the thickness of one sample (DORefA2) was determined to be ~ 230 nm (Fig. 2B, top), with atomic force microscopy (AFM) used to characterize film roughness (Figure S5). Using Eq. (1), assuming a refractive index of around ~ 1.56^26^, the 0^th^, 1^st^, and 2^nd^ order of reflection of this film was estimated to be at ~ 1435, 479, and 287 nm respectively. This was verified using UV–VIS–NIR reflectance spectrophotometry, reflectance between 200–400 nm (a combination of the 1st and 2nd order of reflection) was noted and broad IR reflectivity above ~ 1400 nm (Fig. 3A). Characterizing the optical properties of each isoform fabricated under the same conditions revealed the same three conserved optical features; UV reflectivity at ~ 200–300 nm, visible reflectivity at ~ 350–450 nm (often combined with the UV peak), and broad IR reflectivity spanning the near- and short-wave IR region up to 3300 nm (Fig. 3B). As expected, SORef1 peak reflectivity was slightly higher than other samples (~ 480 nm). These differences may be due to slight variations in purity, solubility, and refractive index. Controlling the optical properties in the near- and short-wave IR regions is of particular interest to those in the defense industry as these include commonly surveilled electromagnetic (EM) regions which are often targets for EM signature reduction technologies^27^. By varying the angle of incidence, the angle-dependent reflectivity of these single-layer films was then characterized (Fig. 4). As the angle of incidence increased from 20° to 70°, there appears to be a small blue-shift due to thin-film interference (Fig. 5A), whilst the normalized reflectance of all samples at 350–450 nm reduces from ~ 0.6 to ~ 0.25. Concomitantly, normalized reflectance between 650 and 750 nm increased from ~ 0.25 at 20° to ~ 1 at 70°. The result is a shift in peak reflectance in the visible region from 350–450 nm to 650–750 nm (Fig. 5B). When characterizing silicon substrates alone, increasing the angle of incidence appears to cause reflectance intensity below 1500 nm to increase and reflectance intensity above 1500 nm to decrease, but overall there is a conservation of reflectivity across the spectrum (Figure S6). With a (230 nm) reflectin layer, however, this changes as increases in the angle of incidence now result in a decrease in the intensity below 450 nm and an increase in the intensity above 450 nm (slightly higher for SORef1). Reflectivity at 650–750 nm therefore becomes the peak reflectance in the visible region at larger angles of incidence. Notably, a peak emerges at ~ 2370 nm, which we attribute to the Si wafer. The emergence of this peak appears to have been magnified by the reflectin layer. This angular dependent reflectivity can have negative implications, especially for emerging sensing/anti-camouflage technologies^28^, and may therefore represent a hindrance to the integration of reflectin-based Si films/coatings into modern bio-based camouflage technologies.

**Figure 6:**
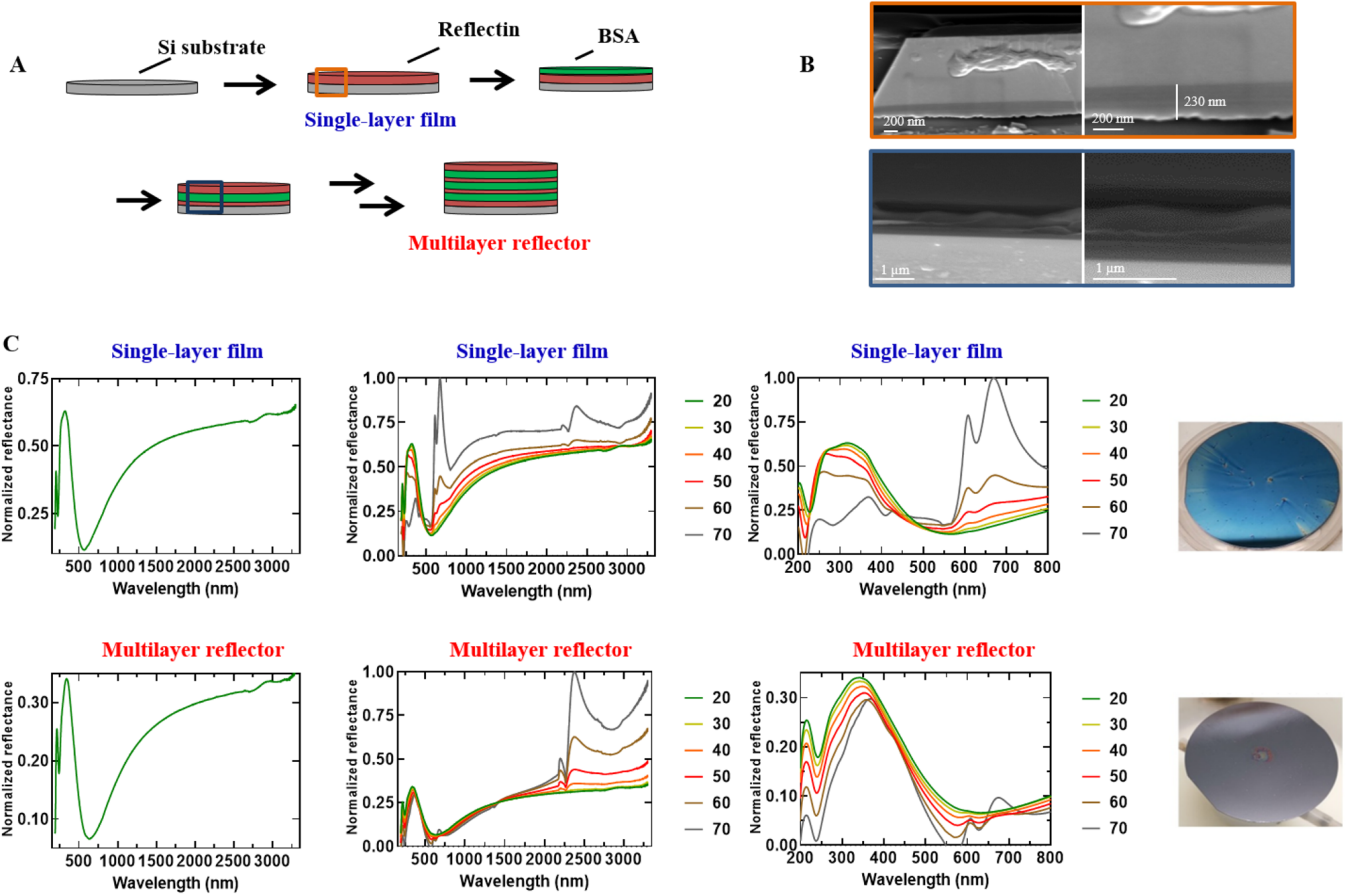
(**A**) Schematic figure showing the fabrication of reflectin single- and multilayer thin-films (**B**). (**C**) UV–Vis–NIR reflectance spectra of reflectin DORefA2 single-layer film (top) and DORefA2 Bragg reflector (bottom) along with the corresponding camera image. Left column; full spectra at 20°, middle column; full spectra at 20°–70°, right column; UV–Vis reflectance spectra at 20°–70°. Right: Optical images of DoRefA2 single-layer film (top) and DoRefA2/BSA multilayer (bottom).

**Figure 7:**
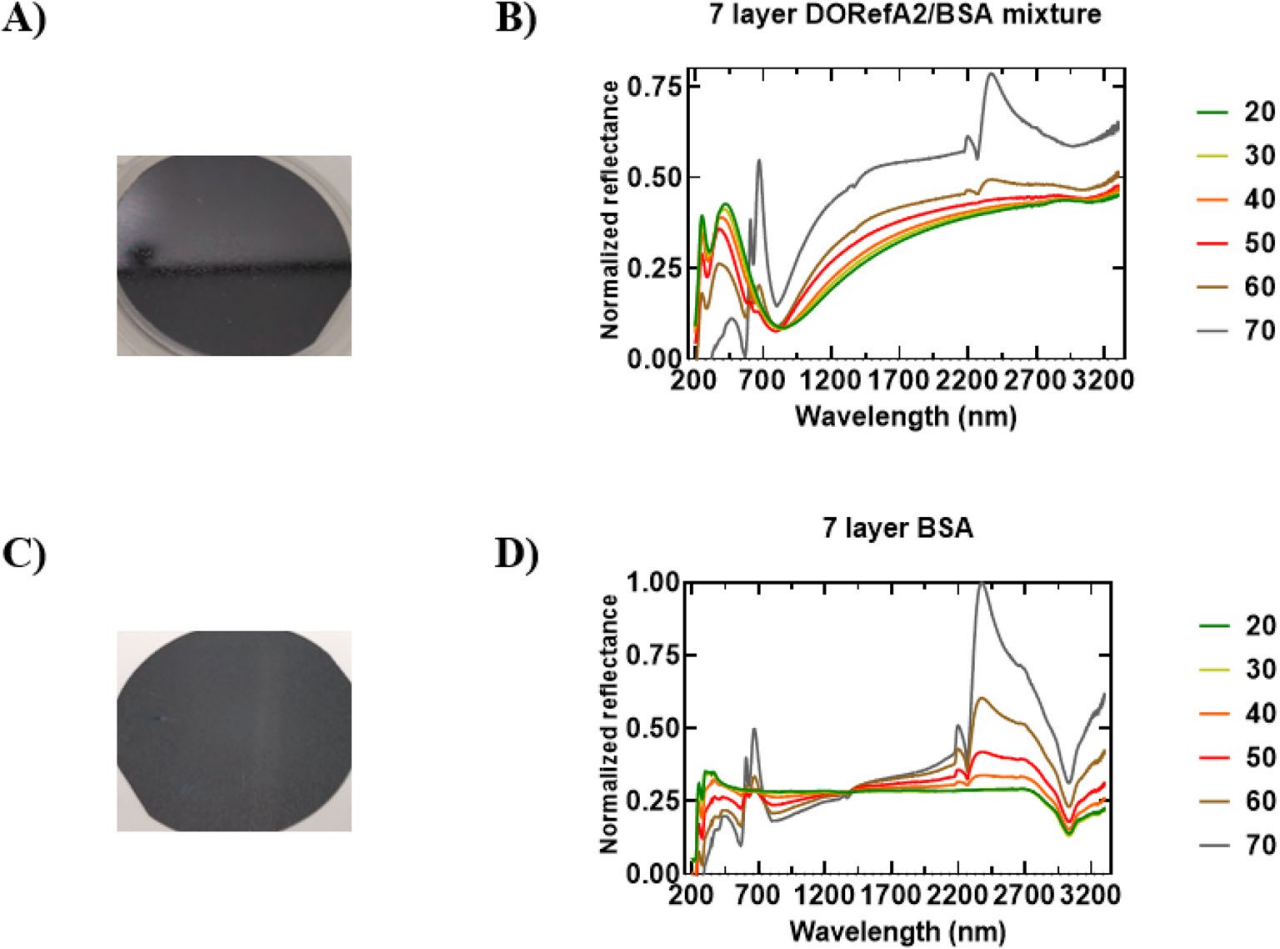
(**A**) Optical image of DORefA2/BSA 7 layered film following fabrication by spin coating 7 layers of a 1:1 mixture of DORefA2 (1% w/w in HFIP):BSA (1% w/w in HFIP) onto a clean Si wafer. Camera images were taken following drying. (**B**) Corresponding UV–Vis–NIR reflectance spectra (185–3300 nm). The angle of incidence was varied between 20° and 70° (10° intervals). (**C**) Optical image of BSA 7 layered film following fabrication by spin coating 7 layers of BSA (1% w/w in HFIP) onto a clean Si wafer. Camera images were taken following drying. (**D**) Corresponding UV–Vis–NIR reflectance spectra (185–3300 nm). The angle of incidence was varied between 20° and 70° (10° intervals).

Taking more direct inspiration from cephalopods, we report our initial findings revealing that a reflectin-based multilayer reflector composed of alternating layers of reflectin and bovine serum albumin (BSA, refractive index: 1.602)^29^—a stable and readily available protein – leads to films with conserved peak reflectance in the visible region at all angles of incidence. BSA in this case mimics the presence of the extracellular space, which separates the multiple reflectin-filled lamellae in iridophores (Fig. 1B). Using spin coating, a seven-layered film was fabricated by coating reflectin and BSA sequentially, allowing the film to dry for a few minutes prior to the application of the next layer (Fig. 6A). An odd number of layers was selected to ensure both the top and bottom layer was comprised of reflectin protein, and we found that 7 layers allowed for multilayer effects to become pronounced. Cross-sectional SEM was used to investigate layering, with SEM on an intermediate tri-layered film suggesting that well-defined layers are not clearly observed, possibly due to some mixing between layers (Fig. 6B). Markedly, at 20°, while all spectral features of our single-layer films were maintained (Fig. 6C, left panel), increasing the angle of incidence now had little to no impact on reflectivity below 1500 nm (Fig. 6C, middle panel). As the angle of incidence increased from 20° to 70°, normalized reflectance intensity was mostly conserved and the visible peak at ~ 350–450 nm was retained as the peak reflectance in the visible region (Fig. 6C, right panel). As the SEM suggests that well-defined layers are not observed, we sought to investigate whether this unique optical response is the result of a single layer composed of a mixture of reflectin and BSA, or is due to some pseudo-multilayered structure that is reliant on our method of sequential coating. Controls fabricated using a DORefA2/BSA mixture and BSA alone (Fig. 7A–D respectively) still exhibited angle-dependent reflectivity below 1500 nm, suggesting that both layer separation and composition, respectively, are significant factors that contribute to angle-independent visible reflectivity. However, further characterization, including precise determination of layer thickness, is required to confirm this suggestion. Interestingly, for our multilayer film, at wavelengths above 1500 nm we noted that the increase in reflectance intensity was amplified, with the same peak at around ~ 2370 nm emerging at 60°/70°, but to an even greater extent. To our knowledge, this is the first example of a reflectin-based film whose specular reflectance has been shown to be modulated with respect to viewing angle. Thus, combined with the dynamic capabilities of the current generation of reflectin-based materials, such as reconfigurable IR reflectivity^12,13^, moving towards more biomimetic configurations may enable the design of next-generation dynamic, optically active, angle-independent materials. Based on theoretical considerations^5^, the reduced angular dependence exhibited by our multilayered reflector may be explained by the morphology of the reflectin layers, with SEM micrographs of reflectin layers revealing the formation of irregular, wrinkled microstructures upon coating (Figure S7). These irregular configurations are thought to arise due to stresses applied during the spin coating process and have been shown to impart unique optical properties^30^, but further analysis and characterization is required to clarify this mechanism.

**Figure 8:**
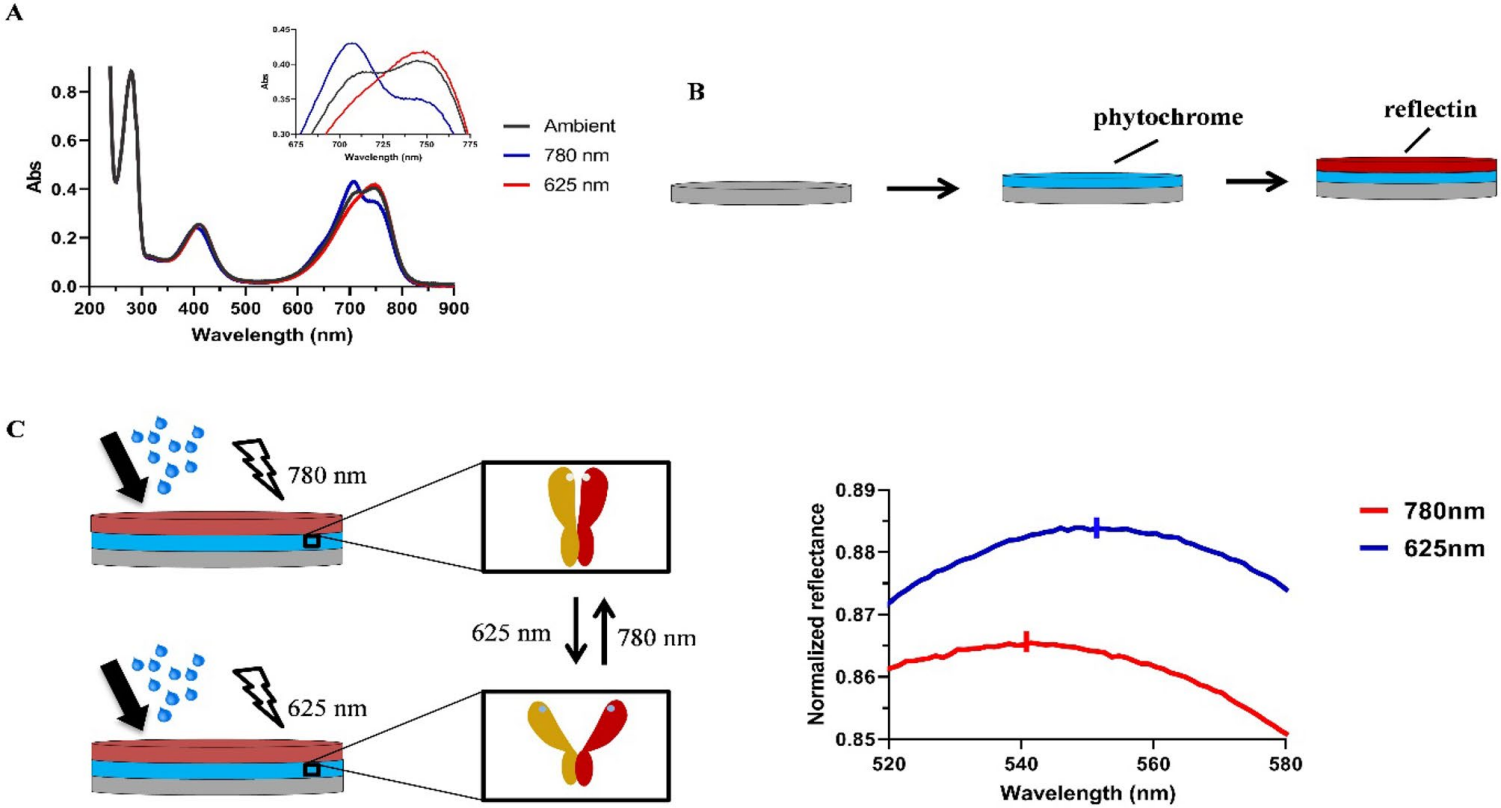
(**A**) UV/Vis spectra of *A. fabrum* phytochrome in H_2_O after being exposed to ambient, red (625 nm), and far- red (780 nm) light. (**B**) Schematic figure showing the integration of phytochromes into reflectin films using spin-coating. (**C**) Schematic figure showing proposed mechanism of phytochrome switching between ‘open’ and ‘closed’ states in response to activation, alongside the corresponding peak reflectance.

**Figure 9:**
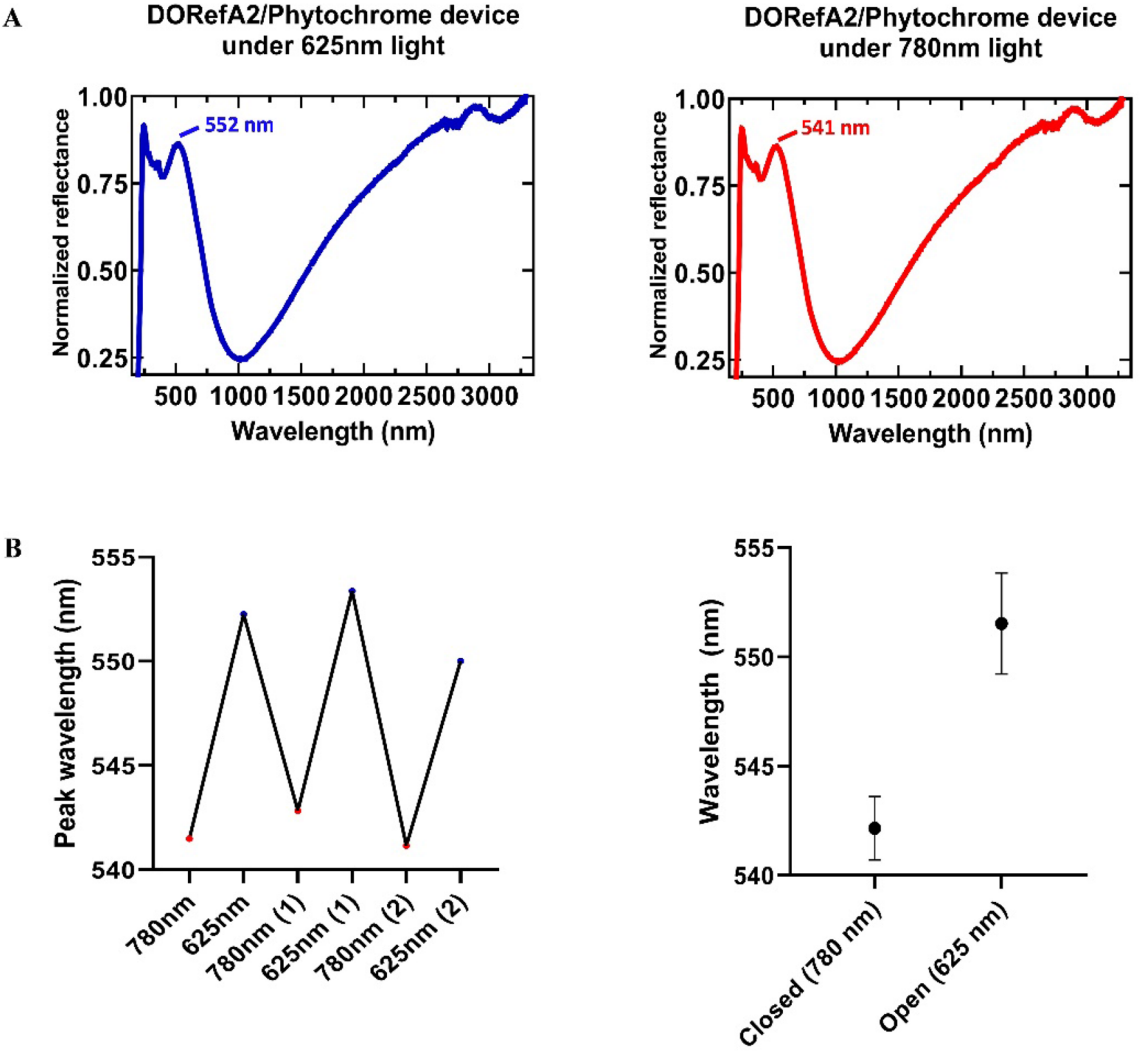
(**A**) DORefA2/phytochrome device full spectra upon exposure to 625/780 nm light in the presence of water vapor. (**B**) Left: illustration of cycling of the DORefA2/phytochrome film between a peak reflectance of λ =  ~ 541 nm and λ =  ~ 552 nm. The experiment was repeated until the film demonstrated degradation. Right: mean and SD plotted.

Designing multilayer films provided the opportunity to investigate the incorporation of other proteins into our materials. Of interest is the use of novel methods to control the optical properties of reflectin thin-films. The phytochrome family of photoreceptor proteins is an attractive target as they undergo a reversible light-induced conversion between two states which involves a conformational change in the protein^31,32^. The use of light to modulate the properties of materials is a well-defined field^33^, for example the development of photochromic liquid crystalline materials which offer a range of dynamic functions such as photoinduced phase transitions, photoalignment, photomobility, and phototunable reflection^34,35^. In solution, after being exposed to 625 nm light, the UV spectra of phytochrome 1 from *Agrobacterium fabrum* (Figure S8) was found to contain a peak around 750 nm, which corresponds to an ‘open’ state of the protein. After exposure to 780 nm light the peak shifted to ~ 710 nm, corresponding to a ‘closed’ state of the protein (Fig. 8A). A reflectin/phytochrome device was then designed and subsequently fabricated by spin coating a layer of phytochrome onto a clean Si wafer, allowing the film to dry, and spin coating a layer of reflectin (Fig. 8B). By monitoring the peak reflectance, we found that long exposure to 625/780 nm light in the presence of water vapor resulted in changes to the optical properties of the device, which we hypothesise are attributed to fluctuations in the thickness of the phytochrome layer (Fig. 8C), although this will require further in-depth study to definitively rule out other possible explanations such as reversible vapour-induced morphological perturbation. Continuous exposure to 625 nm light in the presence of water vapor resulted in a peak reflectance of ~ 552 nm, while exposure to 780 nm light in the presence of water vapor resulted in a blue-shift to ~ 541 nm. We demonstrated the reversibility of this process by cycling between the two states (Fig. 9), representing the basis for a possible novel, reversible method of controlling the optical properties of reflectin thin-films. This dynamic optical activity results from changes occurring in the phytochrome layer, evidenced by our observation of similar shifts in single layered phytochrome films (Figure S9) and phytochrome/BSA films (Figure S10), and the absence of such in reflectin bi-layers (Figure S11). Notably, the spectral features of reflectin films are conserved following integration of phytochromes, although incorporating reflectin into phytochrome films appears to have slightly reduced the wavelength shift. This wavelength shift is nevertheless comparable to photochromic devices designed by Gorodetsky and coworkers^11^, and, if combined with the properties of multilayer reflectin-based films may lead to a new class of angle-independent photoinducible reflectin-based camouflage devices.

In conclusion, characterizing reflectin-based single layer films has revealed previously unreported optical properties conserved across all samples, such as UV reflectivity and broad reflectance in the near/short-wave IR regions. The angle-dependent reflectivity of single-layer films has been determined, revealing significant spectral shifts associated with changes to the angle of incidence. Moving towards a more biomimetic configuration in our initial investigations led to reduced angle-dependence while maintaining the spectral features of single-layer films, and if examined further may represent a step towards developing angle-independent reflectin-based camouflage technologies. Finally, the integration of phytochrome visible light-induced isomerism into reflectin-based films is described, conserving the optical properties of reflectin films but enabling small pertubations of peak reflectance, adding to the catalogue of methods to control reflectin-based materials optical properties post-fabrication.

## Materials and methods

### Protein expression and purification

The genes encoding reflectins were digested by NdeI and XhoI and ligated into either a pETM11(+) vector (containing a kanamycin resistance gene) or a PMA-T vector (containing an ampicillin resistance gene) digested by the same restriction enzymes, yielding pETM11_XXRefYY/PMa-T_XXRefYY, where XX corresponds to the origin of the reflectin gene, and YY corresponds to the reflectin isoform. Vectors (pETM11_ESRef1a, pETM11_ESRef2D, pETM11_DORefA2, pETM11_ESRef2A, and pETM11_SORef1 were transformed into BL21(DE3) cells (Novagen) and expressed at 37 °C using Overnight Express Instant Terrific Broth (TB) or LB media (Formedium) supplemented with 30 μg mL^−1^ kanamycin/50 μg mL^−1^ ampicillin. All isoforms were completely insoluble when expressed in *E. coli* and were sequestered within inclusion bodies. Inclusion bodies were resuspended in buffer A (20 mM Tris HCl, 100 mM NaCl), sonicated (5 × 15 s, 45 s intervals, 40%), and centrifuged (21,000 g, 30 min). The pellet was then resuspended in wash buffer (20 mM Tris HCl, 5 mM EDTA, 5 mM DTT, 2 M Urea) supplemented with 2% Triton and centrifuged (21,000 g, 30 min). This Triton wash was then repeated, followed by centrifugation (21,000 g, 30 min) and pellet resuspension in wash buffer. After a final centrifugation step (21,000 g, 30 min) the pellet was solubilized in denaturing buffer (6 M guanidine hydrochloride), adjusted to pH 8 using NaOH, and left stirring overnight. The sample was filtered through 5, 0.4, and 0.2 µm sterile filters, clarified by centrifugation (48,000 g, 60 min) and purified using HPLC on an Agilent 1260 Infinity system using a reverse phase C18 column. Elution conditions were: 95% Buffer A: 5% Buffer B to 5% Buffer A: 95% Buffer B at a flow rate of 5–25 mL min^−1^ over 20 min (Buffer A: 99.9% water, 0.1% trifluoroacetic acid; Buffer B: 99.9% acetonitrile, 0.1% trifluoroacetic acid). The pure fractions were pooled, flash-frozen in liquid nitrogen, and lyophilized. Protein purity was assessed by SDS-PAGE on 4–12% Bis–Tris precast gels (Bio-Rad, USA).

### Transformation and plasmid preparations

Plasmid DNA (1 μL) was mixed with 25–50 μL of *E. coli* competent cells and the mixture placed on ice for 30 min. The sample was heat-shocked at 42 °C for 10–30 s and placed on ice for 5 min. SOC medium (500–950 μL) was added at room temperature and cells incubated at 37 °C for 45–60 min. The cells were spread onto a kanamycin (50 μg mL^−1^) or ampicillin (30 μg mL^−1^) antibiotic-selective plate and incubated overnight at 37 °C. For DNA amplification, transformations were performed using DH5α competent cells, while for protein expression BL21(DE3) was used. A single colony from each plate was used to inoculate 10 mL of LB medium supplemented with kanamycin (30 μg mL^−1^) or ampicillin (50 μg mL^−1^). The starter cultures were incubated at 37 °C and 180 rpm overnight. The culture (500 μL) was mixed with 500 μL of a sterile 50% glycerol solution, flash-frozen in liquid N_2_, and stored at -80 °C. For plasmid preparations, QIAGEN spin miniprep kit was used following the recommended protocol. DNA concentrations were determined using the NanoDrop 2000.

### UV spectroscopy

Phytochrome samples were dissolved in MilliQ H_2_O at 0.5 mg mL^−1^ and incubated overnight. UV/vis spectra were collected using a Cary 60 UV–Vis spectrophotometer (Agilent technologies) from 200 to 900 nm.

### Atomic force microscopy

Atomic force microscopy (AFM) imaging was carried out in contact mode using an Asylum Research MFP-3D (Oxford Instruments, High Wycombe, UK) atomic force microscope using a NuSense Scout 350 cantilever (NuNano, Bristol, UK). AFM data were analysed with the Gwyddion software package, http://gwyddion.net/.

### Scanning electron microscopy

SEM was carried out on reflectin-based films using a FEI Quanta 250 microscope with accelerating voltage of 5–8 kV. Samples were sputter-coated with 5 nm Au/Pd to enhance electrical conductivity.

### Reflectance spectrophotometry

The reflectance spectra of 1% reflectin thin-films were collected at room temperature using an Agilent Cary 5000 series UV–VIS–NIR spectrophotometer with a variable angle specular reflectance accessory (VARSA). Spectra were collected between 185 and 3300 nm with a scan rate of 600 nm min^−1^ (1 nm data interval, average time 0.1 s). The angle of incidence was varied between 20° and 70° (10° intervals). All spectra were collected relative to a Si wafer background and normalized relative to the highest reflectance peak.

### Spin coating

Reflectin samples were dissolved in hexafluoroisopropanol (HFIP) at 1% (w/w). Silicon wafer substrates were cleaned with piranha solution (3:1 of sulfuric acid and 30% hydrogen peroxide, hazardous solution) for 1 h, rinsed with HPLC-grade water, polished using lens tissue, rinsed onto the substrates before spin coating at 1500 rpm for 60 s. Films were allowed to dry in a fume hood at room temperature. Reflectin samples on glass microscope slides were fabricated in the same way as Si wafers. A2/BSA multilayer films were fabricated by first spin-coating a 1% (w/w) sample of DORefA2 in HFIP onto a silicon wafer, and once dry, further spin coating a 1% (w/w) sample of BSA in HFIP. This process was then repeated 2.5 times to yield a multilayer film with seven alternating layers; A:B:A:B:A:B:A, where A is DORefA2 and B is BSA. Reflectin/phytochrome thin-films were fabricated by first spin-coating phytochrome (5 mg mL^−1^in HFIP/H_2_O) onto a Si wafer, followed by spin coating DOrefA2 (1 mg mL^−1^in HFIP).

### Photoisomerism

Reflectin/phytochrome samples were continuously exposed to LED light (625 or 780 nm) for 3 min in a dark room while water vapor was applied to the surface for 2 s, at 30 s intervals. Water vapor is applied by holding the film over a water bath which was heated to 50 °C for 2 s.

## Supplementary Information

Supplementary Figures.
