# Supplementary material for: Design and fabrication of recombinant reflectin-based multilayer reflectors: bio-design engineering and photoisomerism induced wavelength modulation
Source: Sci Rep. 2021 Jul 16;11:14580. doi: 10.1038/s41598-021-94042-6 (PMC8285536; doi:10.1038/s41598-021-94042-6)
Supplement: Supplementary file 1 — Supplementary Figures. [file 41598_2021_94042_MOESM1_ESM.docx]

**Supplementary Information**

**Design and fabrication of recombinant reflectin-based multilayer reflectors: bio-design engineering and photoisomerism induced wavelength modulation**

*Emmanuel Wolde-Michael^1^, Aled D Roberts ^l^, Derren J Heyes ^l^, Ahu G Dumanli ^2^, Jonny J Blaker ^2^, Eriko Takano ^l^, Nigel S Scrutton ^l^ **

^l^EPSRC/BBSRC Future Biomanufacturing Research Hub, Manchester Institute of Biotechnology, Department of Chemistry, The University of Manchester, Manchester, M1 7DN

^2^Department of Materials & Henry Royce Institute, The University of Manchester, Manchester, M13 9PL

*** Email: Nigel.Scrutton@manchester.ac.uk

**Supplementary Figures**

**
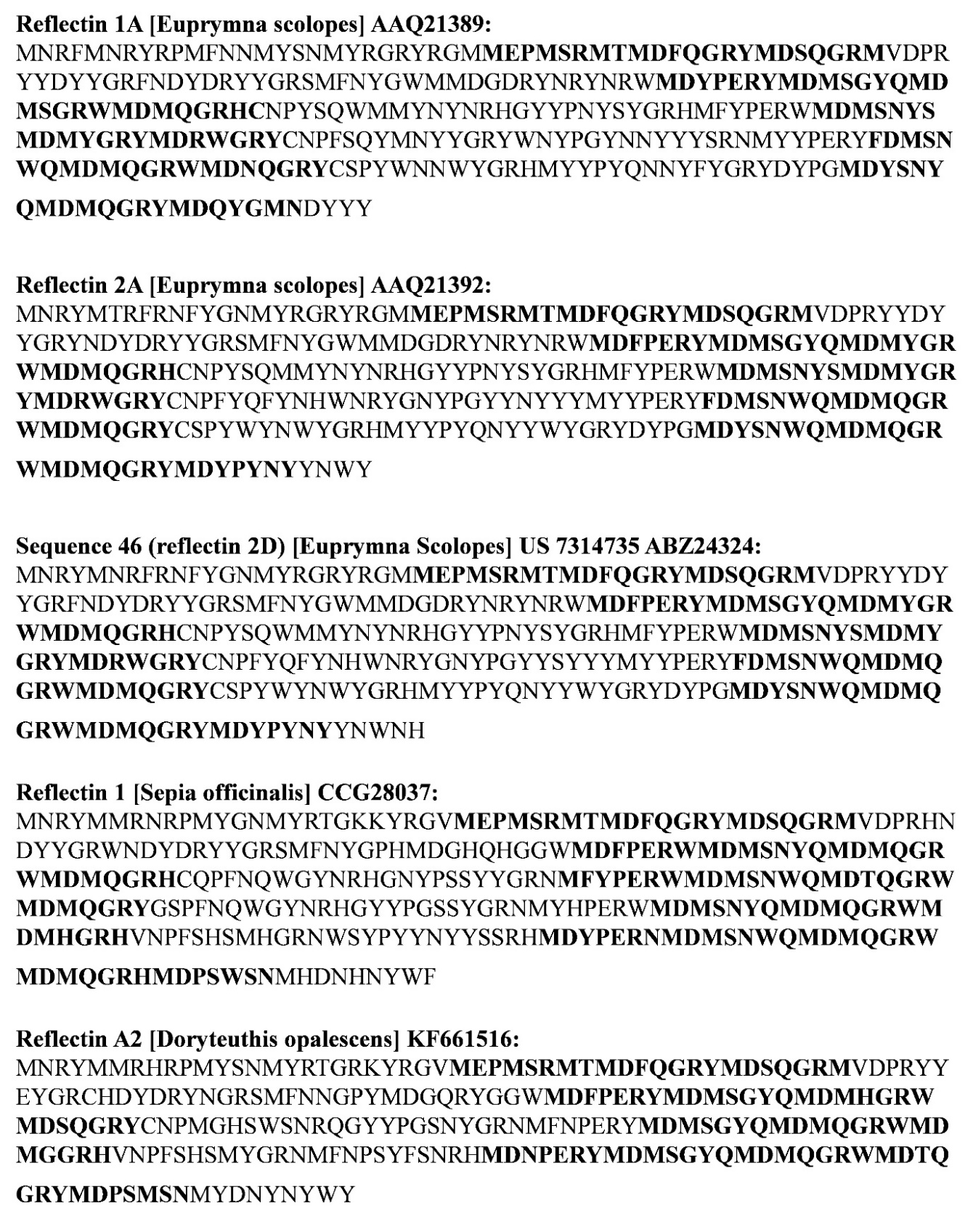
**

**Figure S1** Name, origin, UniProt ID, and amino-acid sequence of all reflectin isoforms used in this study. Repeating motif regions are highlighted in bold.


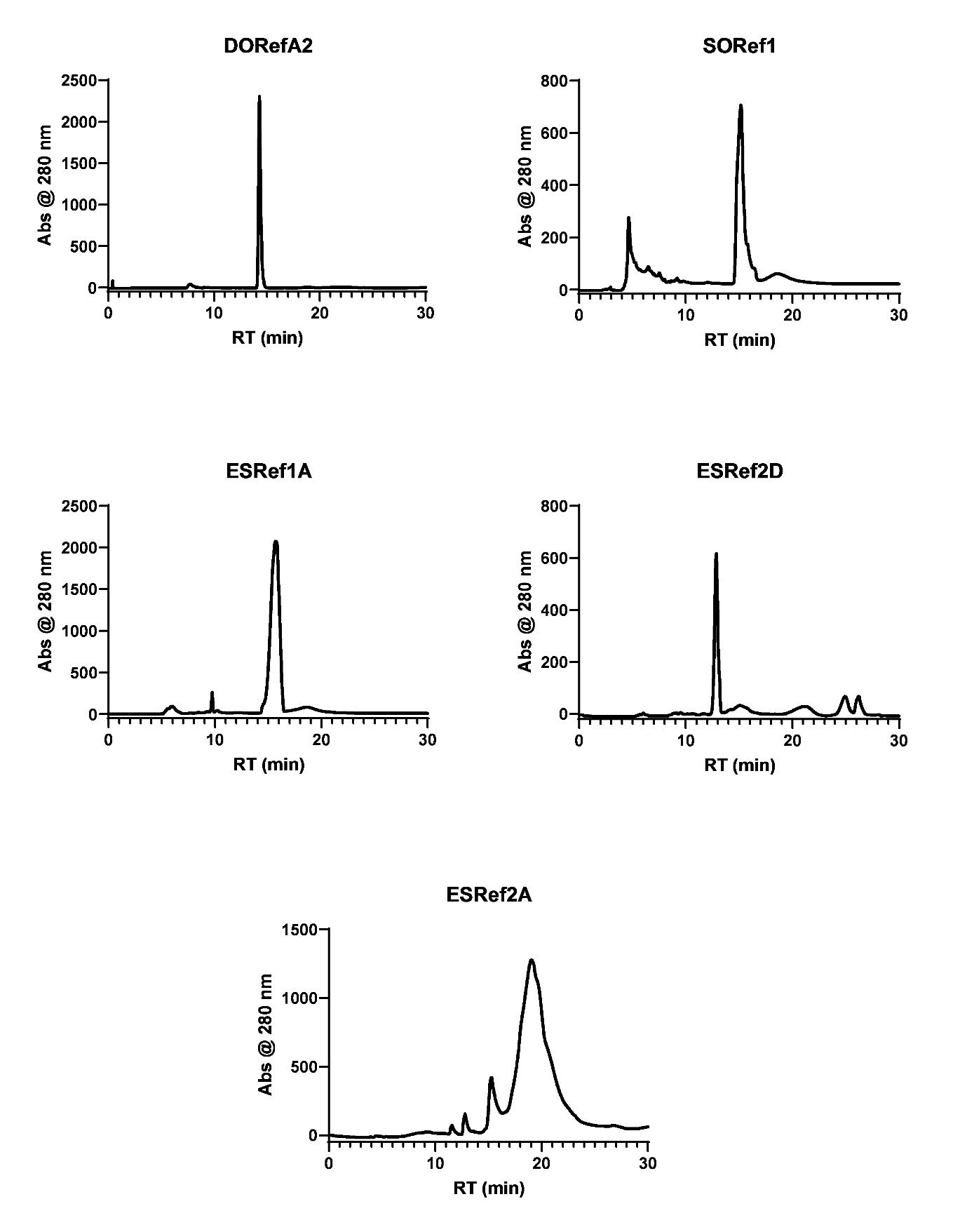


**Figure S2** HPLC chromatograms of purified reflectin proteins eluted with 95:5 Buffer A:Buffer B to 0:100 Buffer A:Buffer B over 30 minutes (Buffer A: H_2_O + 0.1% TFA, Buffer B: ACN + 0.1% TFA).


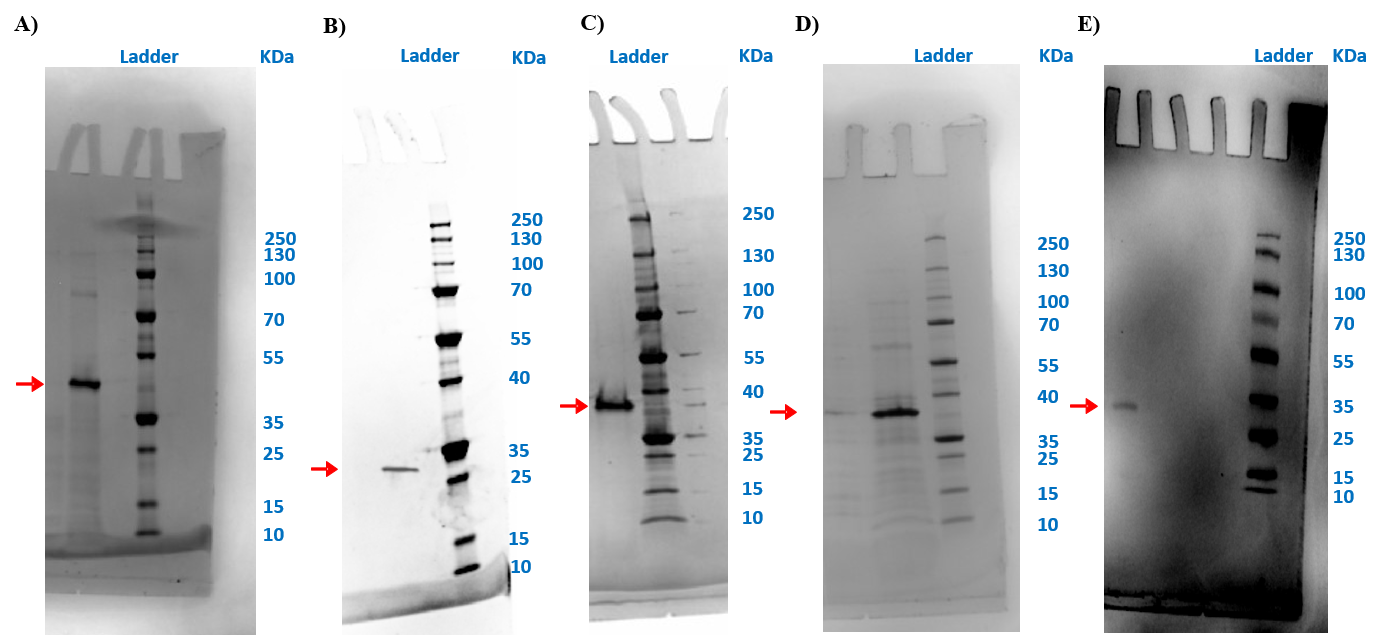


**Figure S3** SDS PAGE gel analysis of purified reflectin proteins A) ESRef2D, B) DORefA2, C) ESRef2A, D) ESRef1A, E) SORef1. The arrow highlights the band of each protein.


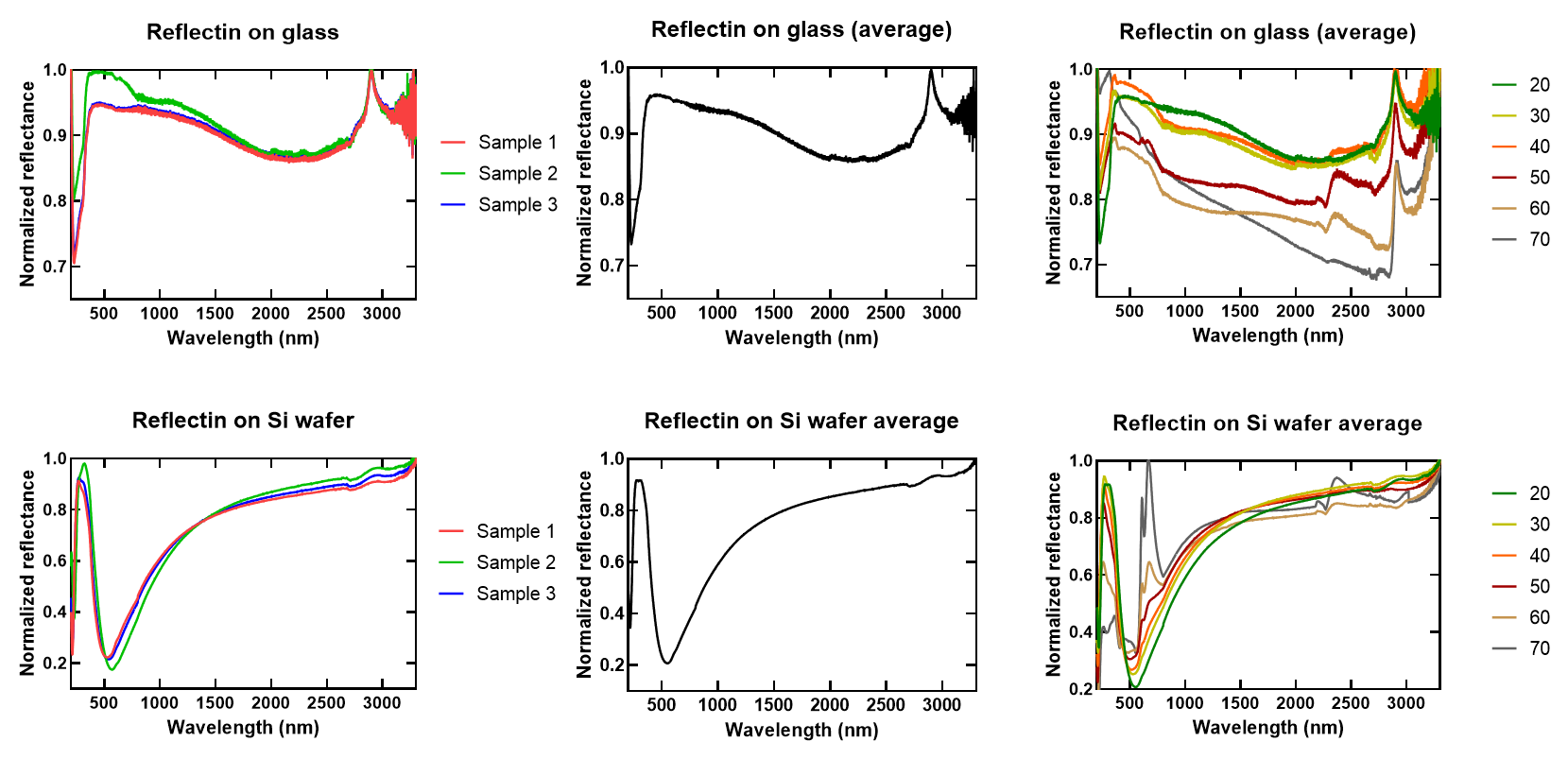


**Figure S4** Left: UV-VIS-NIR reflectance spectra of DORefA2 films on glass and Si wafers at 20 degrees. The spectra are the average of 3 individual repeats (technical replicates gave almost identical spectra) and have been recorded for 3 separate samples to illustrate reproducibility. Middle: Average UV-VIS-NIR reflectance spectra of DORefA2 films on glass and Si wafers at 20 degrees. Right: Average UV-VIS-NIR reflectance spectra of DORefA2 films on glass and Si wafers at various angles between 20-70 degrees.


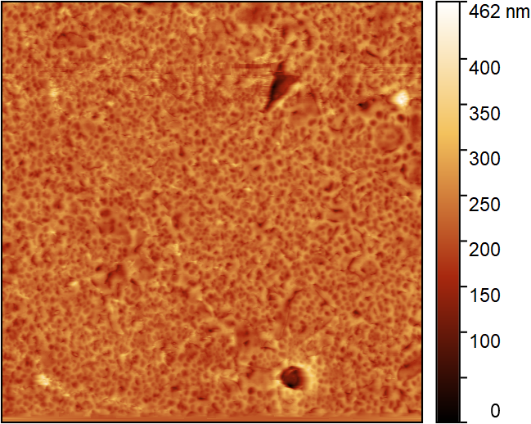

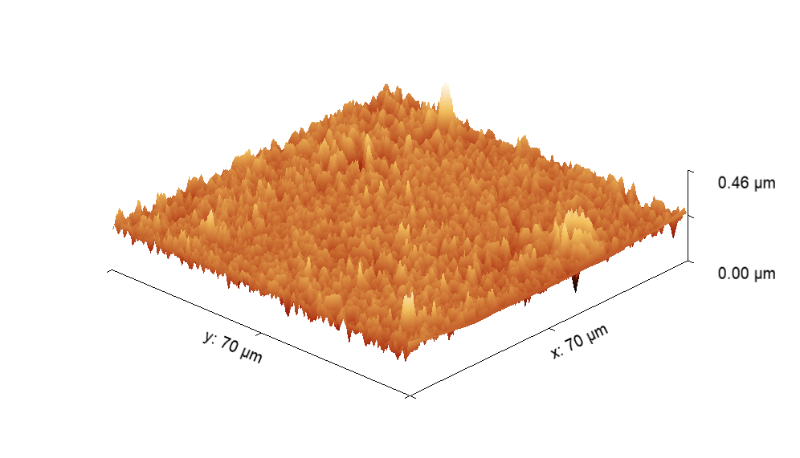


**Figure S5** Representative atomic force microscopy (AFM) images of a single-layered DORefA2 thin-film. The AFM data was processed using the Gwyddion software package, <http://gwyddion.net/>.


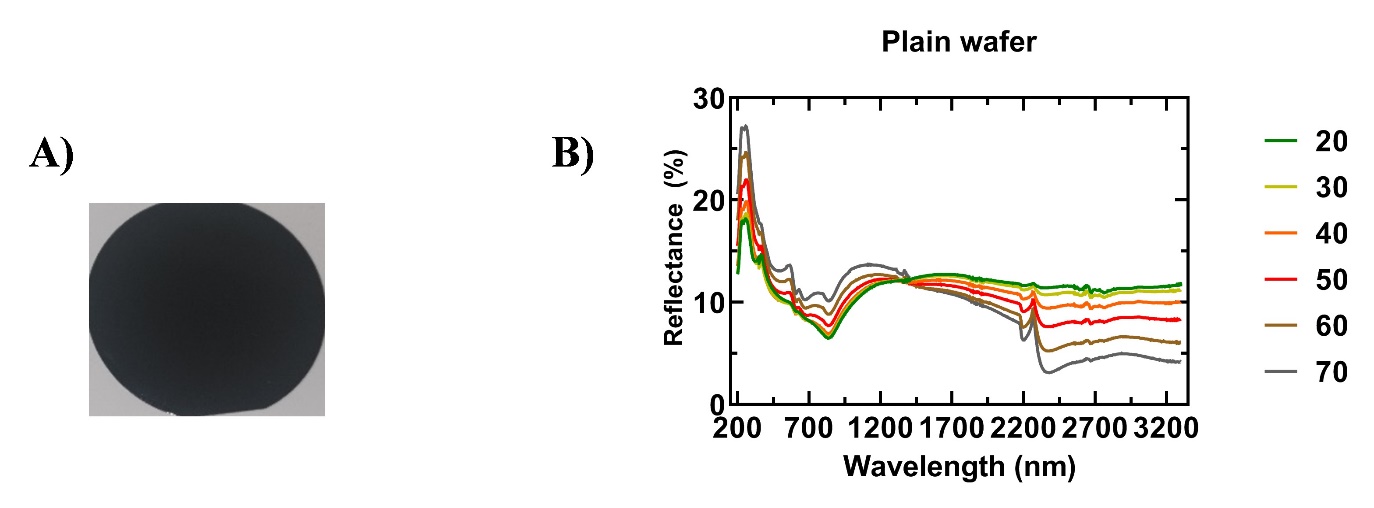


**Figure S6** A) Optical image of a clean dry Si wafer following etching with pirahna solution. B) Corresponding UV-Vis-NIR (background) reflectance spectra (185-3300 nm). The angle of incidence was varied between 20-70 degrees (10 degree intervals).


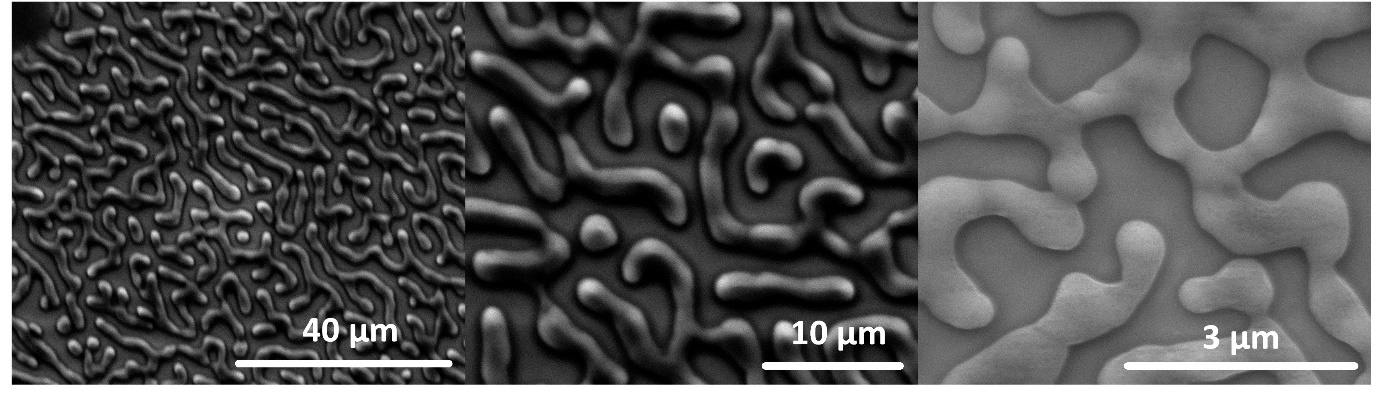


**Figure S7** Representative SEM micrographs of the surface of a reflectin thin-film fabricated by spin coating 1% w/w reflectin in HFIP onto a clean Si wafer.


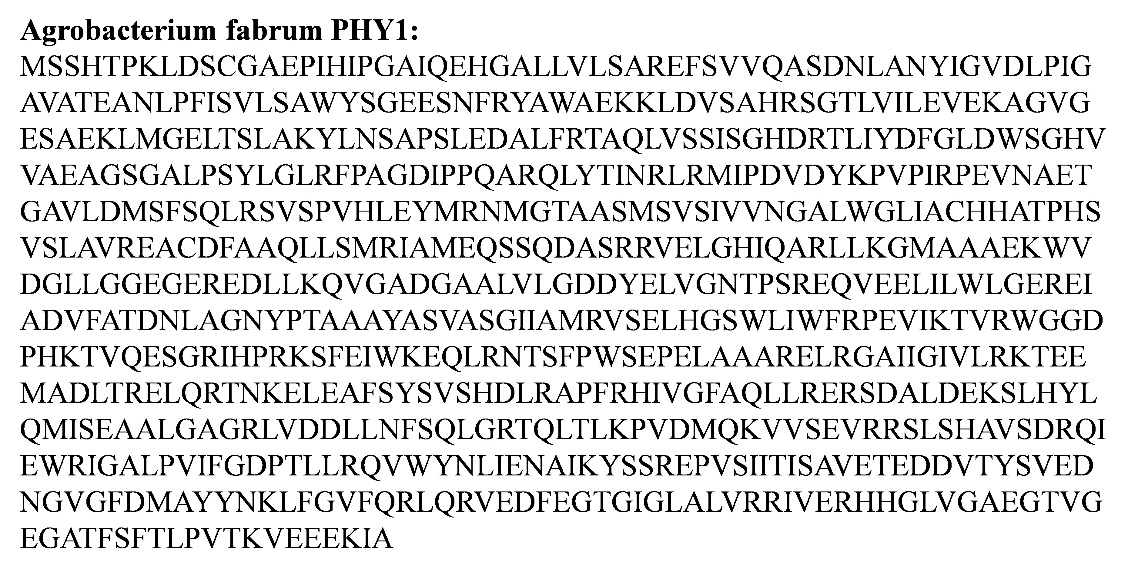


**Figure S8** Amino acid sequence of phytochrome 1 from *Agrobacterium fabrum* used in this study.


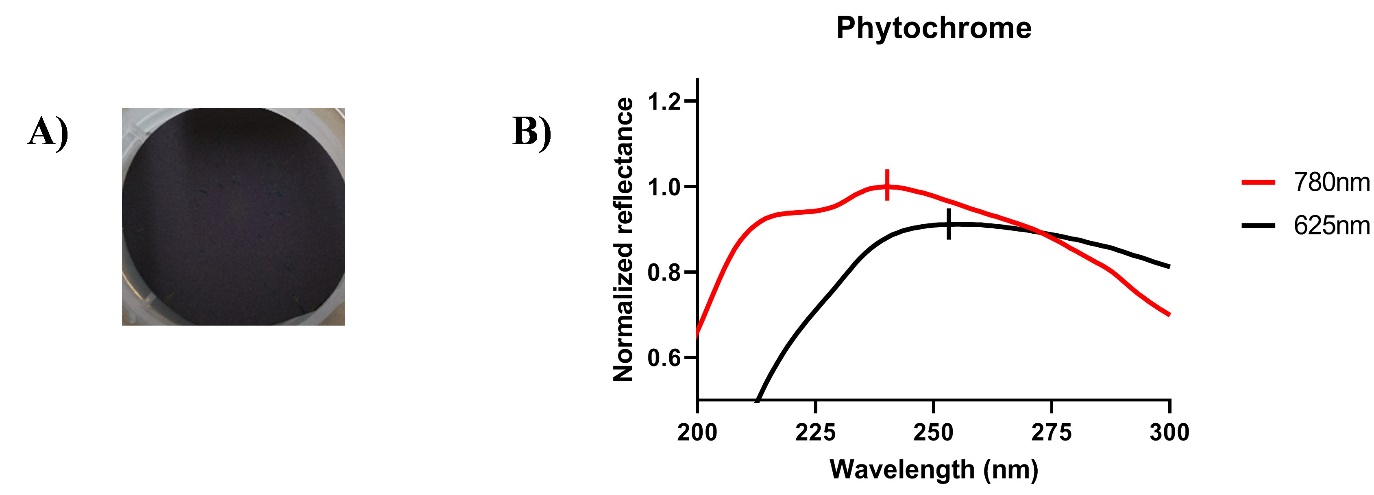


**Figure S9 A)** Optical image of a phytochrome single-layer film following fabrication by spin coating the protein (5% w/w in HFIP/H_2_O) onto a clean Si wafer. Camera images were taken following drying. **B)** Corresponding reflectance spectra after illumination with 625 nm and 780 nm light in the presence of water vapour. Peak reflectance shifts from 240 nm when exposed to 780 nm light to 255 nm when exposed to 625 nm light.


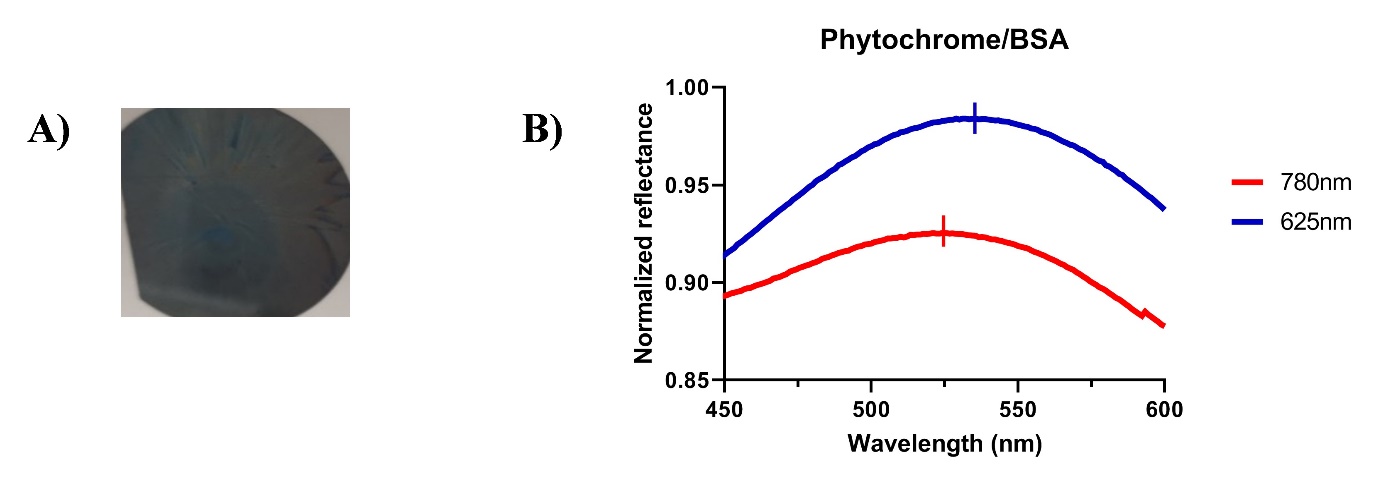


**Figure S10 A)** Optical image of phytochrome/BSA film following fabrication by spin coating BSA (1% w/w in HFIP) followed by phytochrome (5% w/w in HFIP/H_2_O) onto a clean Si wafer. Camera images were taken following drying. **B)** Corresponding reflectance spectra after illumination with 625 nm and 780 nm light in the presence of water vapour. Peak reflectance shifts from 524 nm when exposed to 780 nm light to 532 nm when exposed to 625 nm light.


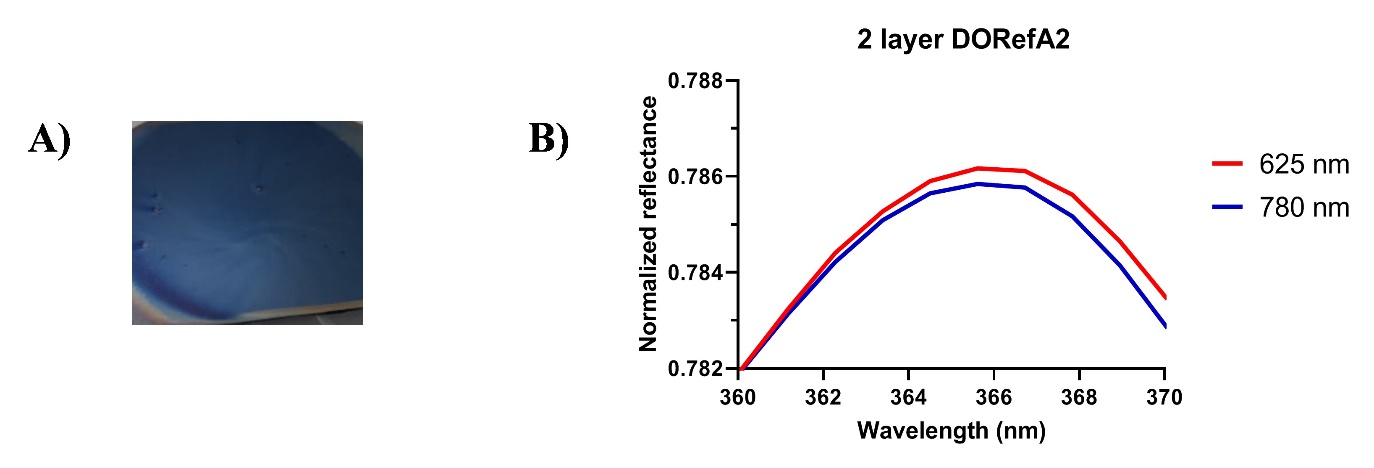


**Figure S11 A)** Optical image of DORefA2 2 layered film following fabrication by spin coating 2 layers of DORefA2 (1% w/w in HFIP) onto a clean Si wafer. Camera images were taken following drying. **B)** Corresponding reflectance spectra after illumination with 625 nm and 780 nm light in the presence of water vapour. Peak reflectance is maintained at ~366 nm.
